# New Insights from Metabolomics in Pediatric Renal Diseases

**DOI:** 10.3390/children9010118

**Published:** 2022-01-17

**Authors:** Simona Riccio, Maria Sole Valentino, Antonio Paride Passaro, Marica Izzo, Stefano Guarino, Emanuele Miraglia del Giudice, Pierluigi Marzuillo, Anna Di Sessa

**Affiliations:** Department of Woman, Child and General and Specialized Surgery, University of Campania “Luigi Vanvitelli”, 80138 Naples, Italy; simonariccio92@gmail.com (S.R.); mariasolevalentino@gmail.com (M.S.V.); antoparidepassaro@gmail.com (A.P.P.); maricaizzo@gmail.com (M.I.); stefano.guarino@policliniconapoli.it (S.G.); emanuele.miraglia@unicampania.it (E.M.d.G.); pierluigi.marzuillo@gmail.com (P.M.)

**Keywords:** metabolomics, renal, disease, children

## Abstract

Renal diseases in childhood form a spectrum of different conditions with potential long-term consequences. Given that, a great effort has been made by researchers to identify candidate biomarkers that are able to influence diagnosis and prognosis, in particular by using omics techniques (e.g., metabolomics, lipidomics, genomics, and transcriptomics). Over the past decades, metabolomics has added a promising number of ‘new’ biomarkers to the ‘old’ group through better physiopathological knowledge, paving the way for insightful perspectives on the management of different renal diseases. We aimed to summarize the most recent omics evidence in the main renal pediatric diseases (including acute renal injury, kidney transplantation, chronic kidney disease, renal dysplasia, vesicoureteral reflux, and lithiasis) in this narrative review.

## 1. Introduction

Metabolomics represents the latest ‘omics’ branch in the tangled field of pediatric renal diseases [1,2]. Omics sciences (including genomics, transcriptomics, proteomics, metabolomics) have recently become recognized as the most innovative approach for the identification of novel biomarkers of various diseases (such as diabetes, obesity, and nephropathy), both in adults and children [3,4,5,6]. In fact, the molecular approach of these branches allows expanding knowledge in a refined manner, because of the dynamic role of different metabolites in the functional and regulatory pathways of distinct diseases [7,8]. In particular, kidney disease, as one of the most significant emerging chronic condition worldwide, is becoming a challenging target of therapeutic development [1,2,9]. Moreover, the deepest pathophysiological knowledge from metabolomics studies might provide insightful perspectives on the wide spectrum of renal diseases (ranging from neonatal conditions, to acute and chronic kidney injury, cystic diseases, and congenital anomalies of the kidney and urinary tract (CAKUT)) at an early age [1,10,11]. Given that, the complex management of these conditions might take advantage of these innovative approaches through the identification of noninvasive biomarkers.

In view of the advances in this field, in this narrative review, we aimed to summarize the most recent evidence on metabolomics in pediatric renal diseases.

## 2. Metabolomics in AKI 

The spectrum of potential dramatic consequences in newborns diagnosed with acute kidney injury (AKI) represents a great challenge for researchers. 

AKI, previously referred to as acute renal failure (ARF), represents a frequent and serious problem in hospitalized children [12,13,14], with high rates of mortality and morbidity [15,16]. The prevalence of AKI varies from 5% among hospitalized patients to 30–50% in patients who undergo cardiac surgery and those in intensive care units [17,18]. Approximately 1–5% of ARF patients require dialysis [19]. Prematurity represents an independent risk factor for AKI, because of an incomplete nephrogenesis and an increased susceptibility to hypoperfusion, characterized by high renal vascular resistance, high plasma renin activity, low glomerular filtration rate, and decreased sodium reabsorption by the proximal tubules. In addition, in the postnatal course, premature newborns often presented with hypotension, hypoxia, and need of cardiorespiratory support [20].

In the clinical setting, AKI is defined as a 50% or greater increase in serum creatinine (sCr) from baseline. However, sCr is an unreliable and insensitive indicator of altered kidney function in the early phase. In fact, sCr concentrations may not change until about 50% of kidney function has already been lost. Furthermore, sCr does not accurately represent kidney function until a steady state has been reached [21]. As demonstrated, early intervention might be fundamental in preventing the pathophysiological events leading to AKI [16,18,22]. From this perspective, the identification of candidate biomarkers might positively impact on AKI diagnosis and consequences [23].

As an early AKI biomarker in preterm infants, urinary Neutrophil gelatinase-associated lipocalin (uNGAL) concentrations have been widely studied in preterm infants [24,25,26,27].

Significantly increased levels of urinary NGAL, osteopontin (OPN), and cystatin C (CysC) in preterm infants, developing AKI and decreased levels of epidermal growth factor (EGF) and uromodulin (UMOD) have also been found to be able to predict AKI [28]. Moreover, urinary concentrations of a protein, namely liver fatty acid-binding protein (L-FABP) and kidney injury molecule-1 (KIM 1), have been demonstrated as further candidate biomarkers of AKI [29,30,31]. Notably, uNGAL has also been found to be, not only a good screening test for the early AKI diagnosis, but also a predictor of mortality and severity of HIE in asphyxiated neonates [32].

Although there is good evidence for ‘old’ AKI biomarkers [33,34,35,36,37], the use of the most innovative techniques such as omics allowed identifying ‘new’ potential biomarker for AKI in children (Table 1).

Promising results in the analysis of urinary polypeptides were also derived from newborns with ureteropelvic junction (UPJ) obstruction [38].

Mercier et al. first examined the urinary metabolomic profiles of preterm neonates with AKI [39]. Urine samples from infants on the second day of life were analyzed using broad-spectrum nuclear magnetic resonance (NMR) metabolomics. A clear distinction between the profiles of neonates with and without AKI was reported. Hippurate and homovanillate differentiated AKI from non-AKI profiles. In particular, higher homovanillate and lower hippurate levels were found in these subjects. Metabolomics was also able to differentiate the urinary profiles of neonates with and without AKI diagnosis, and metabolic profiles correlated with gestational age. Gestational age correlated, not only with metabolites of aminoacyl-t-RNA biosynthesis in newborns without AKI, but also with metabolites of pyruvate metabolism in patients with AKI, as well as propylene glycol and ethylene glycol. For all infants, threonine was significantly linked to gestational age [39].

### New Potential Biomarkers in Children with AKI

Liu et al. discovered serum IL-6 and IL-8 as early biomarkers of AKI in children undergoing cardiac surgery [40]. Furthermore, among patients with AKI, high IL-6 levels were associated with prolonged mechanical ventilation, suggesting that circulating cytokines in patients with AKI may have deleterious effects on other organs, including the lungs [40].

Nguyen et al. assessed urinary aprotinin levels in pediatric subjects undergoing CPB, by indicating urinary aprotinin excretion as an early biomarker of AKI [41].

Devarajan et al. in a proteomic study conducted on 30 children undergoing cardiac surgery, demonstrated that urinary α1-microglobulin, α1-acid glycoprotein, and albumin represent early, accurate, inexpensive, and widely available biomarkers of AKI in children who developed AKI after cardiac surgery. They also offer prognostic information on duration of AKI and length of hospitalization after surgery [42].

Beger et al. performed a metabolomics analyses of urine samples obtained from 40 children that underwent cardiac surgery using CBP, for correction of congenital cardiac defects [23]. Urine samples were obtained from each patient prior to surgery and at 4 h and 12 h after surgery. Twenty-one children presented with AKI at 48–72 h after cardiac surgery. A dopamine metabolite, such as omovanillic acid sulfate, was proposed as a sensitive AKI biomarker [23].

Wang et al. collected urine samples from 27 septic children with AKI and 30 septic children without AKI, to harness urinary metabolic profiling to discover potential biomarkers of septic acute kidney injury in pediatric patients in intensive care units [43]. The authors used ultra-performance liquid chromatography-quadrupole time-of-flight mass spectrometry (UPLC-QTOF/MS) for profiling and multiple regression analysis to explore the potential biomarkers of sepsis with AKI. They identified a clear distinction in the UPLC-QTOF/MS results for septic children with and without AKI after the development of sepsis, specifically 18 and 17 metabolites with different levels at 12 and 24 h, respectively. Metabolic pathways associated with septic AKI included lipid metabolism, particularly processes involving glycerophospholipid metabolism. L-Histidine, DL-indole-3-lactic acid, trimethylamine N-oxide, and caprylic acid were uncovered as potential biomarkers of septic AKI at 12 h, while gentisaldehyde, 3-ureidopropionate, N4- acetylcytidine, and 3-methoxy-4-hydroxyphenylglycol sulfate were identified as potential candidates at 24 h. Of note, a combination of metabolites was more effective as a diagnostic marker than individual metabolites [43].

In a recent study by Muhle-Goll et al., the diagnostic accuracy of NMR urine metabolite patterns for the diagnosis of neonatal and pediatric AKI according to the kidney disease improving global outcomes (KDIGO) definition was investigated [44]. A cohort of 65 neonatal and pediatric patients (0–18 years) with established AKI of heterogeneous etiology were compared, both to a group of apparently healthy children (n = 53), and to a group of critically ill children without AKI (n = 31). A panel of four metabolites were identified for AKI diagnosis. In particular, urinary citrate levels were significantly reduced, whereas leucine and valine levels were elevated [44].

**Table 1:** Main findings of omics studies in children with AKI.

References	Study Design and Methods	Population (n)	Main Findings
Whang et al. [43]	Prospective, case-control study; UPLC-QTOF/MS	27 septic children with AKI and 30 septic children without AKI	A metabolic set-up differentiating children with or without AKI was found
Nguyen et al. [41]	Prospective, case-control study; SELDI-TOF-MS.	106 patients −74 without AKI (mean age of 4.5 ± 5.3 yr.) −32 with AKI (mean age 3.6 ± 5.9 yr.)	Urinary aprotinin was an early predictor of AKI and adverse outcomes.
Devarajan et al. [42]	Prospective, case-control study; SELDI-TOF MS.	30 children undergoing −15 AKI (mean age of 4.0 ± 7.8 yr.) −15 controls (mean age 3.9 ± 5.3 yr.)	Urinary α1-microglobulin, α1-acid, glycoprotein, and albumin represent early and accurate biomarkers of AKI after cardiac surgery
Beger et al. [23]	Prospective, case control study; UPLC/MS analysis MS/MS analysis	40 children: −19 without AKI (mean age 4.3 ± 4.8 yr.) −21 with AKI (mean age 2.7 ± 3.7 yr.)	Urinary HVA-SO4 was a sensitive and predictive biomarker of AKI after pediatric cardiac surgery
Muhle-Gall et al. [44]	Prospective, case-control study; NMR spectroscopy	65 children with AKI, 53 healthy children, and 31 critically ill children without AKI.	A panel of four metabolites allowing AKI diagnosis was found.

UPLC: ultra-performance liquid chromatography; MS: mass spectrometry; MS/MS: Tandem mass spectrometry; HVA-SO4: homovanillic acid sulfate; CIN: contrast-induced nephropathy; SIRS: systemic inflammatory response syndrome; WB: Western blot; L-FABP: Liver fatty acid-binding protein; BWs: birth weights; KIM-1: kidney injury molecule-1; NAG: N-acetyl-b-D glucosaminidase; MMP-9: matrix metalloproteinase-9; UPLC-QTOF/MS: ultra-performance liquid chromatography-quadrupole time-of-flight mass spectrometry; SELDI-TOF-MS: surface-enhanced laser desorption/ionization time-of-flight mass spectrometry; B2M: beta2-microglobulin; Cys C: cystatin C; UMOD: uromodulin; CBP: cardio-pulmonary bypass; NMR: nuclear magnetic resonance.

## 3. Metabolomics in Kidney Transplantation

Non-invasive technologies to monitor kidney allograft health utilizing high-throughput assays of blood and urine specimens are emerging out of the research realm and slowly becoming part of everyday clinical practice [45]. The need for biomarkers in kidney transplantation stems from various observations [46]. First, both early allograft and early patient survival have improved dramatically over the past 40 years (above 90% at 1-year post-transplant), both in adults and in children, but no improvements in longer term outcomes have been reported [47]. Secondly, previous studies reported that molecular and cellular events of acute rejection (AR) occurred before the rise of the most common clinical biomarker, such as sCr [48,49]. Notably, a lack of specificity for diagnosis and identification of a non-rejection kidney injury (NRKI) has been observed for sCr, leading to a need for kidney biopsy in selected cases [50]. In addition, differentiating a superimposed AR from chronic injury progression becomes very difficult in case of patients with elevated sCr from chronic allograft dysfunction and underlying fibrosis. Therefore, kidney biopsy remains the existing gold standard, although it represents a challenging test, especially in childhood, because of its limited feasibility at this age [51]. On this ground, identification of novel and more suitable biomarkers for kidney transplantation (including AR, chronic kidney allograft damage, and NRKI) is required [52].

In recent years, the field of transplantation has seen an increasing number of omic-based studies (which include genomics, epigenomics, proteomics, transcriptomics, and metabolomics); this area has the potential to lead to the development of personalized treatment depending on individual molecular risk profiles [53]. Blydt-Hansen et al. [54] evaluated the utility of urinary metabolomics for noninvasive diagnosis of T cell-mediated rejection (TCMR) in pediatric kidney transplant recipients, in 57 patients with surveillance or indication for kidney biopsy. A defined urinary metabolite profile was correlated to TCRM risk. In particular, 10 out 134 analyzed metabolites, including Proline, PC.aa.C34.4, Kynurenine, and Sarcosine, were found to be the most important in the class prediction [54]. Moreover, among these metabolites, 5–10 resulted as shared with borderline tubulitis, suggesting that the allograft injury associated with T cell-mediated alloimmune response might occur on a continuum of severity. Furthermore, since borderline TCMR was also associated with progressive allograft function decline and failure, metabolomic biomarkers may be more effective for quantifying alloimmune injury than allograft histology [54].

Regarding TCMR, Mincham et al. [55] described urinary changes in CXCL10 and metabolite TCMR discriminant score (MDS) as biomarkers independently linked to TCMR in 49 pairs of sequential kidney biopsies from a single center cohort of pediatric kidney transplant recipients. Urinary CXCL10:Cr and TCMR MDS were obtained at each biopsy and were tested for association with changes between biopsies in acuity, estimated GFR, and 12-month ΔeGFR. Samples were screened to identify sequential biopsy pairs and two biopsies were performed (‘biopsy 1’ performed ≥2.5 months post-transplant, and ‘biopsy 2’ after 21–95 days for follow-up of rejection, protocol, or for new indication). Using each biopsy pair for comparison, ΔeGFR was not found to predict change in acuity. By contrast, the latter was significantly associated with change in urinary CXCL10:Cr and MDS between biopsies [55]. The 12-month ΔeGFR was not predicted by TCMR acuity or CXCL10:Cr at Biopsy 2, but an inverse correlation was observed with urinary MDS. Moreover, changes in eGFR poorly correlated with evolving TCMR acuity on histology [55].

In another 2017 prospective study [56], Blydt-Hansen et al. investigated the utility of urinary biomarkers for early noninvasive detection of antibody-mediated rejection (ABMR) in patients with (n = 40) and without (n = 278) ABMR. The ABMR score was associated with the presence of donor specific antibodies, biopsy indication, and some of the Banff Lesion scores, specifically ct, t, ah, and cg scores (which are used to evaluate respectively tubular atrophy, tubulitis, arteriolar hyalinosis, and glomerular basement membrane double contours in renal transplant biopsies [57]) and retained accuracy when applied to subclinical cases. Exploratory classifiers segregating samples based on concurrent TCMR identified overlapping metabolite signatures between ABMR and TCMR, by suggesting a similar pathophysiology of tissue injury [56].

Chronic kidney allograft damage is characterized by interstitial fibrosis and tubular atrophy (IFTA) and glomerulosclerosis (GS), and associated with progressive functional decline and premature allograft failure. Landsberg et al. [58] identified metabolomic classifiers linked to eGFR, IFTA severity, and percentage of GS; similarly to histologic findings, urinary metabolite profiles identifying IFTA and GS severity were distinct from those associated with eGFR. Moreover, the addition of some clinical features, such as months post-transplant (GS, IFTA) and proteinuria, GFR, and age (GS only), improved prediction. It was shown that, even when applied to potentially confounding phenotypes (for example alloimmune inflammation and/or AKI), the combined clinical and metabolomic classifier effectively identified severity of GS and IFTA. Based on the findings of previous studies on the utility of metabolomics to allograft injury [54,59,60] and chronic kidney disease (CKD) stage [61] assessment, this study provided the first evidence for metabolomics as a marker for severity of chronic histological changes in allograft CKD.

Notably, metabolomics was also investigated in the field of pediatric non-rejection kidney injury (NRKI). Younger children may be more predisposed to NRKI, in particular if they have received an adult-size kidney transplant; their limited capacity to increase their cardiac output [62] can lead to an inadequate kidney perfusion [63].

In a single-center pediatric cohort study [64], Archdekin et al. extended their previous work for detection of patterns related to allograft kidney injuries using the same set of quantified metabolites for identification of clinically relevant classifier of NRKI independent of acute rejection. Urine samples without rejection were split into NRKI, pre-NRKI, and no NRKI. The NRKI discriminant score distinguished between NRKI from samples with no NRKI and pre-NRKI. In addition, the NRKI score sensitivity was found to significantly discriminate between NRKI and rejection (CMR, AMR, and mixed rejection): in the subset of all samples with elevated serum creatinine, the score was successfully tested for differentiating the two different conditions [64]. Regarding the NRKI score, some metabolites (such as Proline [65] or ADMA [65,66]) have been previously linked to reduced GFR [19]. However, the patterned change in metabolism represented by the entire group of metabolites, rather than each metabolite, was found to be crucial [64].

Taken together, these findings supported an intriguing role for urinary metabolites as a noninvasive tool for rejection and NRKI identification; nevertheless, their use in clinical practice is hindered by some critical issues, such as the lack of universal procedures for study design (i.e., handling and processing of biological samples), the lack of standard guidance for method validation, and the absence of adequate prospective and retrospective cohort studies to allow interlaboratory reproducibility in a large population [67].

## 4. Metabolomics Studies on Renal Dysplasia and ADPKD in Children

Renal dysplasia (RD) is a rare disease but represents one of the main causes of chronic kidney disease in childhood and one of the most frequent underlying pathologies in children requiring renal replacement therapy. RD is usually asymptomatic before the evolution in CKD. Ultrasonography reveals a normal-sized or small kidney with increased echogenicity and either absent or poor corticomedullary differentiation, frequently accompanied by the presence of small cysts. Early diagnosis is difficult because of the lack of biomarkers in the initial period of stable renal function. Prompt diagnosis of renal dysplasia is important, in terms of management and long-term prognosis, due to the risk of future end-stage renal disease [68].

Macioszek et al. for the first time, compared the urinary metabolomic signature of children with renal dysplasia with healthy children [68]. The main changes detected derived from the purine, lipid and amino acid metabolism, glycolysis, the TCA cycle, and the urea cycle. They found 28 metabolites significantly different in renal dysplasia children in comparison to the healthy controls, and nine of these were found to differentiate subjects with normal and reduced eGFR. Increased urinary level of N-acetylasparagine, betaine, uric acid, and hypoxanthine, and decreased levels of TMAO, were observed in patients with renal dysplasia and correlated with a decreased eGFR. In this study, the decreased urinary levels of a few acylcarnitines (6-keto-decanoylcarnitine, dodecanedioylcarnitine, hydroxyisovaleroylcarnitine, hydroxydecanoylcarnitine, nonanoylcarnitine, butyrylcarnitine) were observed as statistically significant in renal dysplasia patients, as compared to the control group. These alterations can be explained by blood accumulation of acylcarnitines, potentially associated with mitochondrial dysfunction. The metabolic alterations related to the glutamine, aconitate, and lactate levels were also observed to be statistically significant in the comparison between renal dysplasia patients and the control group [68].

Autosomal dominant polycystic kidney disease (ADPKD) is the most common inherited kidney disease, affecting approximately 1:400 to 1:1000 live births. The disease is characterized by cysts in the kidneys and extrarenal manifestations (e.g., hepatic cysts, mitral valve prolapse, berry aneurysms) and is a cause of end stage renal disease (ESRD) [69]. Cyst growth and accumulation compressing on renal vasculature activate the renin–angiotensin–aldosterone system (RAAS), which, in combination with the upregulation of vasopressin receptors, was responsible for early hypertension. The early onset of hypertension has been linked to rapid decline in estimated glomerular filtration rate (eGFR) and early-onset ESRD [69].

Baliga et al. studied metabolomic plasma profiling of children with ADPKD [69]. They compared this profile with that of healthy children, to identify the markers and pathways that differ between these two groups and those related to the progression of ADPKD. Amino acid, glucose (including glycolysis, citric acid cycle and gluconeogenesis), fatty acid metabolism, and oxidation were the main metabolic pathways significantly different between the two groups. Pathway analysis revealed that metabolites in the arginine metabolism (urea and nitric oxide cycles), asparagine, and glutamine metabolism, in the methylation cycle and kynurenine pathway, were significantly changed between healthy and children with ADPDK and continued to diverge from the control levels while the disease progressed. A large number of the identified metabolites were known uremic toxins, including 1-methyladenosine, allantoin, asymmetric dimethylarginine (ADMA), dimethylglycine (DMG), guanidinoacetic acid (GAA), homocysteine (HCy), hypoxanthine, indolacetic acid (IAA) and 5-hydroxyindolecateic acid (5-HIAA), kynurenate, monomethylarginine (MMA), quinolinate, trimethylamine oxide (TMAO), and xanthine. Their accumulation in the plasma of ADPKD, as compared to healthy children, was evident, even prior to the decline of GFR. From the ninety-five identified metabolites, thirty-seven were potentially able to differentiate between the healthy and diseased pediatric populations, as identified by ROC analysis. They also identified the metabolites and metabolic pathways primarily involved in the progression of ADPKD in pediatric patients [69] (Table 2).

## 5. Metabolomics in Chronic Kidney Disease (CKD)

CKD is a major epidemiologic problem worldwide, in which renal hypofunction occurs due to irreversible kidney damage [70]. Due to its progressive course, CKD might represent a dramatic condition with severe long-term consequences [71], potentially leading to death [72]. In clinical practice, a common marker for renal function evaluation and CKD diagnosis, both in adults and children, is represented by the glomerular filtration rate (GFR), which in turn is estimated using the serum creatinine concentration [73]. Despite its wide feasibility, it has several limitations, due to creatinine variability caused by different factors, such as tubular secretion, the influence of overall body weight, muscle mass, dietary intake, nutrition, hydration status, and age. Moreover, CKD is often asymptomatic in its early stages, and in the pediatric population often remains underdiagnosed for several years [72]. Given that, the discovery of new biomarkers for early CKD diagnosis might dramatically improve the overall management of the disease. In this scenario, metabolomics might provide novel insights in this field [11,70,72,74] (Table 3).

Metabolomics analyses are typically categorized into two complementary approaches: targeted metabolomics, which analyses a group of defined metabolites related to a specific metabolic pathway; and untargeted metabolomics, which simultaneously analyses as many features as possible after minimal sample treatment, without any prior knowledge [72].

Benito et al. [70] used the former through ion-pairing liquid chromatography quadrupole time-of-flight mass spectrometer (LC-QTOF–MS) to detect potential biomarkers in pediatric CKD, by analyzing plasma arginine–creatine metabolic pathway-related compounds in 56 patients (32 CKD patients and 24 healthy patients). Five metabolites, including glycine, citrulline, asymmetric dymethylarginine (ADMA), and symmetric dimethylarginine (SDMA), were significantly increased in patients with CKD, regardless of their creatinine level, and one metabolite, namely dimethylglycine, which was also increased in patients with high creatinine levels.

Based on LC-QTOF-MS, Benito et al. [72] used untargeted metabolomics to detect other biomarkers for early CKD. In particular, the authors found significant changes in five metabolites. Particularly, four of them (sphingosine-1-phosphate, n-butyrylcarnitine, cis-4-decenoylcarnitine, and an unidentified feature corresponding to a compound with properties similar to 2-DL. aminoadipic acid) were increased in patients with CKD, while one (namely bilirubin) was significantly decreased in these subjects [72].

Targeted metabolomics was also used by Brooks et al. [74] to explore changes in metabolites when passing from a stage 2 to stage 3b of CKD. They identified five metabolites (phosphatidylcoline, tryptophan (Trp), kynurenine (Kyn), creatinine (Cr), and acylcarnitine) and ratios (tyrosine/creatinine (Tyr/Cr), ornithine/citrulline (Orn/Cit), Kyn/Trp, proline/Cit (Pro/Cit), phenylalanine/Trp (Phe/Trp), and SDMA/ADMA) which were significantly different between the cohorts, likely due to the change in kidney function between the two stages [74].

Changes in metabolites underlying CKD progression were also analyzed by Denburg et al. [75]. The research was based on plasma samples of 645 CKD children, aged from 6 months to 16 years, with the purpose of identifying new biomarkers of CKD progression, defined as the requirement of kidney replacement therapy or a 50% reduction in eGFR. The authors recognized 825 metabolites using untargeted metabolomics. In particular, for those with baseline eGFR ≥60 mL/min per 1.73 m^2^, seven metabolites were significantly associated with CKD progression: N6-carbamoylthreonyladenosine, 5,6-dihydrouridine, pesudouridine, C-glycosyltryptophan, lanthionine, 2-methylcitrate/homocitrate, and gulonate. Among those with eGFR <60 mL/min per 1.73 m^2^, higher levels of tetrahydrocortisol sulfate were associated with lower risk of CKD progression [75,76].

As the available therapies after kidney disease onset are currently limited, the early identification of children at risk is crucial to improving their overall quality of life. Sood et al. [77] conducted a population-level cohort study using metabolic profiles from 1,288,905 newborns from 2006 to 2015, to detect the metabolic profiles at birth potentially associated with higher risk of CKD or dialysis. Among the enrolled children, 2086 developed CKD and 641 required dialysis. For CKD, the strongest associations were with citrulline, phenylalanine/glycine, acylcarnitines, acylcarnitine ratios, and ratios between amino acids and acylcarnitines to endocrine markers such as 17-hydroxyprogesterone. For dialysis, the strongest associations were with amino acid ratios (phenylalanine/glycine, phenylalanine/tyrosine, citrulline/tyrosine), acylcarnitine ratios, and ratio of amino acids to acylcarnitine [77].

## 6. Metabolomics in Pediatric Vescicoureteral Reflux

Vescicoureteral reflux (VUR) represents a disease associated with an increased risk of recurrent urinary tract infections and renal scarring [78,79], even if it is still not possible to predict which patients with VUR will develop these complications [80]. Although still limited in childhood, the metabolomics approach is also emerging in this field.

In a case-control study of 2021, Vitko et al. [81] aimed to identify dominant urinary microbiota and metabolic profiles in patients with VUR. For the metabolomic analysis, urine samples were collected from 96 children, including 13 healthy controls, 41 patients with VUR and a history of single urinary tract infection (UTI), and 42 patients with VUR and recurrent UTIs. They focused on particular metabolomics changes associated with bacteria metabolism and inflammatory response in children with VUR, such as changes in glutamate, tryptophan, and histidine degradation pathways [82,83] and specific changes in bile acid metabolism [84]. Moreover, in the same cohort, elevated levels of both amino sugars as components of the bacterial wall and phospholipids as part of a host cell membrane were detected, suggesting a potential bacteria and host cell proliferation in these patients.

Collectively, these metabolomics changes may be potentially helpful in identifying children with VUR at increased risk of recurrent UTIs and renal scarring, although the underlying pathobiology is still poorly defined and the results needed to be validated.

## 7. Metabolomics in Kidney Stone Disease

Lithiasis is a disease with high prevalence and recurrence rates [85], in which metabolic perturbations lead to stone formation in the kidney (nephrolithiasis) or in the urinary tract (urolithiasis), resulting in various clinical manifestations including pain, dysuria, loss of kidney function, decreased bone mineral density, and cardiovascular disease in case of chronic conditions [86]. In the recent years, advances in the knowledge of the role of metabolism in the development and progression of this condition have been reported [87]. In particular, branches of omics such as metabolomics have investigated the change in metabolites linked to lithiasis [88].

Denburg et al. [86] studied the relationship between the composition and function of gut microbial communities and early-onset calcium oxalate kidney stone disease. In particular, the authors, conducted a case-control study on 88 pediatric patients, 44 with kidney stones containing ≥50% calcium oxalate and 44 controls matched for age, sex, and race through untargeted metabolomics. Patients with kidney stones had less diverse gut microbiome, with 31 taxa less abundant with respect to controls, including seven taxa that produce butyrate (*Roseburia* and *Clostridium* species) and three taxa that degrade oxalate (*Enterococcus faecalis*, *Enterococcus faecium,* and *Bifidobacterium animalis*). Moreover, the authors found a difference in 18 metabolites between cases and controls, of whom 10 were more abundant (amino acids and derivatives) and eight were less abundant (lipids and lipid-like molecules). An age dependence of microbial diversity among subjects who were kidney stone formers was also reported, with the lowest diversity among individuals who first formed stones between 9 and 14 years of age.

Wen et al. [88] analyzed the change in metabolites in patients with urolithiasis through serum metabolomics based on ultra-performance liquid chromatography mass spectrometry. The authors investigated a total of 50 patients (30 children with kidney stones and 20 controls) enrolled between January 2018 and July 2019. A statistically significant change in 40 serum metabolites was found in patients with kidney stones involving bilirubin, which was decreased, and molecules involved in retinol metabolism, such as steroid hormone biosynthesis, porphyrin, and chlorophyll metabolism, which were greatly increased [88].

Stone formation may also be linked to genetic causes, as occurs in primary hyperoxaluria type 3 (PH3) [89]. Greed et al. [89] analyzed the urine samples of two children with PH3 through tandem mass spectrometry, to evaluate potential changes in metabolites. The authors found a significantly increase in urine levels of 4-hydroxyglutamate (4OHGlu), the immediate precursor of HOG, in multiple samples from both patients, suggesting this molecule as a reliable marker for PH3 89].

## 8. Limitation of Metabolomics Studies

Although promising, more effort is needed to clarify the potential of metabolomics in pediatric renal diseases. In fact, the findings from these studies have shown some significant differences between serum and urine samples [1,2]. Notably, recent insights have proposed organ biopsy metabolomics to enhance the relevance of data in this attractive field [2,6]. Moreover, it should be noted that metabolomics techniques provide large datasets requiring specialized approaches to data storage, management, and analysis [1,2,6]. Finally, the identification of a significant metabolomics signatures represents a challenging step for all researchers. Therefore, the great potential of metabolomics in the era of personalized medicine needs to be investigated in a comprehensive and multidisciplinary way, to significantly improve both the diagnosis and treatment of pediatric renal diseases [6,7].

## 9. Conclusions

The heterogeneity of renal pediatric diseases and their cardiometabolic burden represents a challenging field for clinicians [7,8]. From this perspective, the recent advances in biotechnological techniques have expanded knowledge on the pathophysiological basis of these conditions [1,2]. In particular, omics approaches (including metabolomics, proteomics, lipidomics, transcriptomics, and genomics) have shown the most promising results, by providing new insights through the identification of potential biomarkers in different diseases [2,4]. Notably, growing evidence has suggested an intriguing role for metabolomics in various kidney diseases, suggesting novel potential biomarkers as meaningful tools for the better management (including diagnosis, treatment, and prognosis) of these conditions [6,7,90]. Taken together, these intriguing findings might have a great impact on the overall quality of life of children diagnosed with renal diseases, but validation is needed in further studies.

## Figures and Tables

**Table 2:** Main findings of omics studies in renal cystic disease in children.

References	Study Design and Methods	Population (n)	Main Findings
Macioszek et al. [68]	prospective, case control study; GC-QQQ/MS, LC-TOF-MS	72 children: −39 with renal dysplasia (mean age 5.68 yr.) −33 healthy controls (mean age 7.28 yr.)	The main changes detected derived from the purine, lipid, and aminoacid metabolism and included glycolysis, TCA cycle, and the urea cycle
Baliga et al. [69]	randomized, double- blind, placebo- controlled phase 3 clinical trial; HPLC–MS/MS	58 patients: 31 undergoing treatment with pravastatin (mean age 16 ± 3 yr.) and 27 with placebo (mean age 15 ± 4 yr.)	Thirty-seven metabolites showed a potential role to differentiate plasma of children with ADPKD and healthy subjects.

GC-QQQ/MS: gas chromatography coupled to triple quadrupole mass spectrometry; LC-TOF-MS: liquid chromatography coupled to time-of-flight mass spectrometry; TCA: Citric acid cycle; HPLC–MS/MS: high-performance liquid chromatography-tandem mass spectrometry.

**Table 3:** Main findings of the omics studies in children with CKD.

References	Study Design and Methods	Population (*n*)	Main Findings
Benito et al. [70]	Cohort study using LC-QTOF-MS based targeted metabolomics of arginine–creatine metabolic pathway to identify potential plasma biomarkers in pediatric CKD	56 patients: −32 patients suffering from CKD aged 3–17 years in different stages of the disease; −24 healthy patients aged 6–18 years	Five metabolites were increased independently of creatinine (glycine, citrulline, ADMA and SDMA) while dimethylglycine was increased when CKD patients had plasma creatinine levels above 12 microg/mL
Benito et al. [72]	Cohort study using LC-QTOF-MS based untargeted metabolomics to identify potential plasma biomarkers in pediatric CKD.	58 patients: −32 patients suffering from CKD aged 3–18 yr. in different stages of the disease; −26 healthy patients aged 6–19 yr.	Four metabolites were increased in patients with CKD (sphingosine-1-phosphate, n-butyrylcarnitine, cis-4-decenoylcarnitine and an unidentified feature with 126.0930 *m*/*z*), while bilirubin was significantly decreased.
Brooks et al. [74]	Cohort study using targeted metabolomics to identify altered biochemical pathways in plasma of adolescents with mild to moderate CKD (stage 2 and 3b).	40 patients subdivided in two cohorts matched by age, gender, and CKD etiology (glomerulopathy and non-glomerularurologic anomalies).	Five metabolites (phosphatidylcholine, Trp, Kyn, creatinine and acylcarnitine) and ratios (Tyr/Cr, Orn/Cit, Kyn/Trp, Pro/Cit, Phe/Trp and SDMA/ADMA) were significantly different between the cohorts.
Denburg et al. [75,76]	Multicenter prospective cohort study using plasma samples of CKD children, enrolled between January 2005 and December 2014, to detect metabolites involved in CKD progression.	645 participants (aged from 6 months to 16 years) with eGFR of 30–90 mL/min per 1.73 m^2^.	825 metabolites were recognized. For children with baseline eGFR ≥60 mL/min per 1.73 m^2^, seven metabolites were significantly associated with CKD progression, such as N6-carbamoylthreonyladenosine, 5,6-dihydrouridine, pesudouridine, C-glycosyltryptophan, lanthionine, 2-methylcitrate/homocitrate and gulonate. Children with eGFR <60 mL/min per 1.73 m^2^ had higher level of tetrahydrocortisol sulfate, which was associated with lower risk of CKD progression.
Sood et al. [77]	Population-level cohort study using metabolic profiles from newborns from 2006 to 2015 to detect metabolic profiles at birth possibly associated with higher risk of CKD or dialysis.	1,288,905 newborns, born between 1 April 2006 and 26 September 2015 for whom newborn screening data were available.	Among the analyzed children, 2086 developed CKD and 641 required dialysis. For CKD, the strongest associations were with citrulline, phenylalanine/glycine, acylcarnitines, acylcarnitine ratios, and ratios between amino acids and acylcarnitines to endocrine markers such as 17-hydroxyprogesterone. For dialysis, the strongest associations were with amino acid ratios (phenylalanine/glycine, phenylalanine/tyrosine, citrulline/tyrosine), acylcarnitine ratios and ratio of amino acids to acylcarnitine.
